# Multiple Protocols Combined with Hyperbaric Oxygen Therapy on the Maintenance of Ovarian Function in Patients After Ovarian Cystectomy

**DOI:** 10.3389/fsurg.2022.877114

**Published:** 2022-05-20

**Authors:** Jie Yu, Yin-Liang Qi, Da-Wei Lu, Qian-Jin Fang, Lan Li, Lin Sang

**Affiliations:** ^1^ Department of Obsterics and Gynecology, The Second People's Hospital of Hefei (Hefei Hospital Affiliated to Anhui Medical University), An Hui, China; ^2^ Department of Hyperbaric Oxygen, Hefei Second People’s Hospital, An Hui, China

**Keywords:** post oophorocystectomy, ovarian reserve function, hyperbaric oxygen, anti-mullerian hormone, ovarian cystectomy

## Abstract

**Objective:**

This study aims to explore the effect of adjuvant hyperbaric oxygen therapy on ovarian function after laparoscopic ovarian cystectomy.

**Methods:**

A total of 60 patients with ovarian cysts treated at our hospital from January 2018 to August 2020 were enrolled. According to the different treatment modalities, the patients were divided into the control and observation groups. Patients in both groups underwent laparoscopic ovarian cystectomy with oral administration of Chinese patent medicine Kuntai capsules after surgery. Hyperbaric oxygen therapy was added to patients in the observation group in addition to the treatment in the control group. The anti-Müllerian hormone (AMH), follicle-stimulating hormone (FSH), luteinizing hormone (LH), estradiol (E2), and antral follicle count (AFC) serum levels were detected in both groups before the operation and at the first and third menstrual cycles postoperatively to evaluate ovarian function.

**Results:**

At the first and third menstrual cycles after surgery, the AMH, E2, and AFC serum levels in the two groups were significantly lower than before surgery, and the FSH and LH serum levels were higher than before surgery. The differences were statistically significant (*P* < 0.05). After the operation, AMH, E2, and AFC serum levels in the observation group were significantly higher than in the control group. FSH and LH serum levels were significantly lower than in the control group, and the differences were statistically significant (*P* < 0.05).

**Conclusion:**

For patients undergoing laparoscopic ovarian cystectomy, the adjuvant hyperbaric oxygen therapy could significantly improve the postoperative ovarian reserve function with remarkable effects.

## Introduction

Ovarian reserve refers to the ability of follicles in the ovarian cortex to grow, develop, and form fertilizable oocytes, and represents the ability of gametogenesis and steroid hormone production in women, including the number of follicles retained in the ovaries and the quality of follicles, with the former reflecting the fertility potential in a woman and the latter determining the age of menopause (1–3). Decreased ovarian reserve function is an early stage of ovarian failure, and early detection, intervention, and treatment can significantly improve the prognosis. Currently, many researchers have found that laparoscopic surgery may affect the ovarian blood flow and function, mainly because the locations of some laparoscopic surgical resections are close to the utero-ovarian ligament or close to the ovarian hilus, which can significantly affect the ovarian blood supply. Meanwhile, the wound surface is prone to oozing blood during laparoscopic surgery, and there are many opportunities for electrocoagulation to stop hemorrhage. Electrocoagulation can cause thermal injury and scarring of ovarian tissue and traumatize the ovarian blood supply (4–6), thereby leading to a decrease in ovarian reserve function. At present, the improvement of ovarian function mainly relies on drugs, such as Kuntai capsules, but some patients still have poor therapeutic effects. Under hyperbaric oxygen, blood oxygen content increases, blood oxygen tension increases, blood oxygen dispersion radius expands, and platelet aggregation and wall attachment in blood vessels are reduced, so as to improve the hypoxia state of peripheral blood vessels, peripheral nerves and gonads, improve the oxygen storage capacity of ovarian tissues, and improve ovarian function (7, 8). In the present study, hyperbaric oxygen was used as adjunctive therapy for patients after laparoscopic ovarian cystectomy, and the results were relatively significant. The details are reported as follows.

## Materials and Methods

### Study Subjects

A total of 60 patients who underwent laparoscopic ovarian cystectomy at our hospital from January 2018 to August 2020 were retrospectively analyzed. The inclusion criteria were as follows: (1) non-pregnant women aged 25–32 years; (2) patients with normal preoperative menstruation and sex hormone levels; (3) patients with unilateral ovarian tumors and were healthy on the opposite side; (4) patients with normal results of the preoperative tumor marker and postoperative pathological confirmation of benign ovarian tumors; (5) patients with no medical or surgical comorbidities or surgical history; (6) patients with no contraindications to surgery. The exclusion criteria were as follows: (1) patients with malignant ovarian tumors; (2) patients with no intraoperative transition to open surgery; (3) patients with pedicle torsion of the ovarian cyst; (4) patients with a history of sex hormone administration; (5) patients with other malignant or gynecological tumors. According to the different treatment modalities, the patients were divided into the control and observation groups. The differences in the baseline characteristics such as age, body mass index (BMI), and tumor diameter were not statistically significant between the two groups (*P* > 0.05), and the data were comparable. The details are illustrated in **Table 1**.

**Table 1:** Comparison of the baseline characteristics between the two groups of patients.

Group	Age (year)	BMI(kg/m^2^)	Diameter of the tumor (cm)
Control group	28.26 ± 2.21	20.82 ± 1.74	5.33 ± 1.87
Observation group	28.10 ± 2.33	20.61 ± 1.79	4.89 ± 2.12
*t*	0.42	0.25	1.03
*P*	0.79	0.80	0.45

### Therapeutic Methods

Laparoscopic ovarian cystectomy was conducted in both groups of patients, and the ovaries were sutured to stop hemorrhage during the operation to avoid electrocoagulation of ovarian tissue. On the second day after the operation, Kuntai capsules were administered orally in both groups of patients at a dose of four capsules three times a day for three months as a course of treatment. On the second day postoperatively, hyperbaric oxygen therapy was added to patients in the observation group in addition to the treatments in the control group. The hyperbaric oxygen therapy protocol was as follows: with the adoption of a multiseat pressurized cabin and a therapeutic pressure of 0.2 MPa, the therapeutic duration was 110 min. It was pressurized for 20 min and stabilized for 70 min. The patients wore a mask to inhale oxygen intermittently after pressure stabilization and rested for 5 min for every 20 min of inhalation, decompressing for 20 min. The hyperbaric oxygen therapy was conducted once daily 10 times as a therapeutic course. The patients rested for 10 days after every two therapeutic courses.

### Observation Indicators

The anti-Müllerian hormone (AMH), follicle-stimulating hormone (FSH), luteinizing hormone (LH), estradiol (E2), and antral follicle count (AFC) serum levels were detected before the operation and at the first and third menstrual cycles postoperatively. The E2, FSH, and LH serum levels were detected by radioimmunoassay, and Shanghai Hengyuan Biotechnology Co., Ltd. provided the detection kit. The AMH serum levels were detected by an enzyme-linked immunosorbent assay, and Nanjing Xinfeng Biotechnology Co., Ltd. provided the detection kit. AFC was detected by an HY-M50 color Doppler ultrasound diagnostic instrument provided by Wuxi Haiying Electronic Medical System Co., Ltd.

### Statistical Methods

SPSS 22.0 software was adopted for the analysis of all data in the present study. The measurement data were expressed as mean ± standard deviation $\left(\overset{¯}{x}\pm s\right)$. A *t*-test was used for pairwise comparisons between groups, and repeated measures of analysis of variance were adopted for repeated measurement data. The countable data were expressed as percentages (%), and the χ^2^ test was adopted for statistical analysis. *P* < 0.05 was considered statistically significant.

## Results

### The Serum Levels of AMH, E2, FSH, and LH

At the first and third menstrual cycles after surgery, the AMH and E2 serum levels in the two groups were significantly lower than before surgery, and the FSH and LH serum levels were higher than before surgery. The differences were statistically significant (*P* < 0.05). The AMH and E2 serum levels at the first and third menstrual cycles were significantly higher in the observation group than in the control group, and the FSH and LH serum levels were significantly lower in the observation than in the control group, and the differences were statistically significant (*P* < 0.05), as shown in **Table 2**.

**Table 2:** The serum levels of AMH, E2, FSH, and LH in the two groups of patients.

		AMH			FSH			LH			E2		
Groups	*N*	Pre-operation	At the first menstrual cycle after surgery	At the third menstrual cycle after surgery	Pre-operation	At the first menstrual cycle after surgery	At the third menstrual cycle after surgery	Pre-operation	At the first menstrual cycle after surgery	At the third menstrual cycle after surgery	Pre-operation	At the first menstrual cycle after surgery	At the third menstrual cycle after surgery
Control group	30	5.71 ± 2.13	0.88 ± 0.34	1.16 ± 0.322	5.36 ± 1.97	15.91 ± 2.42	16.00 ± 2.53	7.71 ± 3.08	23.04 ± 8.82	20.00 ± 2.53	75.05 ± 8.84	54.93 ± 8.87	59.83 ± 8.86
Observation group	30	5.67 ± 1.98	1.15 ± 0.30*	4.06 ± 1.80*	5.77 ± 1.98	12.17 ± 1.95*	9.29 ± 2.10*	7.72 ± 3.03	13.84 ± 4.93*	11.52 ± 4.52*	74.44 ± 8.55	64.89 ± 8.82*	70.01 ± 8.87*

*Note: Compared with the control group,* *P < 0.05.

### The AFC

At the first and third menstrual cycles after surgery, the AFC in the two groups was significantly lower than before the operation, and the difference was statistically significant (*P* < 0.05). The postoperative AFC in the observation group was significantly higher than in the control group, and the difference was statistically significant (*P* < 0.05), as demonstrated in **Table 3**.

**Table 3:** Comparison of the AFC between the two groups of patients.

Groups	Number of cases	Pre-operation	At the first menstrual cycle after surgery	At the third menstrual cycle after surgery
Control group	30	7.70 ± 1.28	5.87 ± 1.52	6.00 ± 1.39
Observation group	30	8.55 ± 1.44	6.63 ± 1.35*	7.66 ± 1.43*

Note: Compared with the control group, *P < 0.05.

## Discussion

As part of the female reproductive organs, the ovary is the most frequent and predisposing site for tumors, which can occur at any age, but tumors mainly occur in the reproductive age. The harm caused by ovarian cysts is well-known. Therefore, for clinically discovered ovarian tumors, surgical treatment should be selected as soon as possible (9). Currently, the treatment of ovarian cysts is mainly surgery (10). However, the impact of surgery on ovarian function has been widely confirmed. Related literature pointed out that the suture method adopted also affects ovarian function (11–13). The above literature suggested that after laparoscopic cystectomy, the postoperative AMH serum level was higher with suture hemostasis than in the electrocoagulation group, and the influence of suture hemostasis on the postoperative ovarian reserve function was less than with the adoption of electrocoagulation hemostasis. The application of sutures during surgery can effectively protect ovarian function. Therefore, for each case with an ovarian cyst, operations should be performed by a senior attending physician to minimize injury to ovarian function. In the present study, the ovarian functions in patients with ovarian cysts were detected after surgery, and it was found that the ovarian function was significantly lower than before surgery, which was consistent with previous studies (14). In addition, some Chinese herbal medicine have been proved to be beneficial for improving ovarian function, such as Kuntai capsules (15). Clinical studies have demonstrated that Kuntai capsules have a protective effect for premature ovarian failure (15, 16). Animal experiments have shown that Kuntai capsules can improve ovarian reserves and fertility by increasing the number of ovarian follicles and inhibiting oocyte apoptosis (17). Moreover, it has been proved that Kuntai capsules can decrease the incidence of follicular atresia and cell apoptosis in mice with impaired ovarian function (18).

A previous study (19) has shown that hyperbaric oxygen therapy for perimenopausal syndrome can significantly improve the perimenopausal clinical symptoms such as insomnia, night sweats, and palpitations. Relevant literature also points out that hyperbaric oxygen plays a role in improving ovarian function. Liu et al. (20) applied hyperbaric oxygen to treat premature ovarian failure. It was found that hyperbaric oxygen could effectively regulate the FSH and LH serum levels in patients, improve clinical symptoms, and shorten the course of the disease. Hyperbaric oxygen therapy for premature ovarian failure could significantly improve the positive regulation of FSH, LH, and E2 serum levels as well as relieve a series of clinical symptoms caused by premature ovarian failure (21). For patients with ovarian pedicle torsion, Svensson et al. (22) found that after laparoscopic reverse torsion and hyperbaric oxygen therapy, a gradual recovery of blood supply in the ovary was confirmed by contrast-enhanced ultrasonography. Li et al. (7) found through an animal study that in rats with chronic stress-induced ovarian dysfunction, hyperbaric oxygen exposure could significantly promote the transduction of the transforming growth factor β1/Smad3 signaling pathway and promote the differentiation and proliferation of the ovarian follicles at all levels, as well as increase hormone levels and restore ovarian function. Wang et al. (8) also pointed out that hyperbaric oxygen exposure could improve ovarian function, increase E2 levels, and increase the weight of the uterus, ovaries, and endometrial thickness through animal models. The results of the present study showed that in the first and third menstrual cycles after the operation, the AMH, E2, and AFC serum levels were significantly lower in both groups of patients than before the operation, and the FSH and LH serum levels were higher than before the operation. These results indicated that laparoscopic ovarian cystectomy could affect ovarian function and lead to decreased ovarian reserve. Meanwhile, the postoperative AMH, E2, and AFC serum levels in the observation group were significantly higher than in the control group, and the FSH and LH serum levels were significantly lower than in the control group. These fully demonstrated that the adjuvant hyperbaric oxygen therapy could regulate the serum hormone levels and improve postoperative ovarian reserve. Adjuvant hyperbaric oxygen therapy could increase blood oxygen content and tension, expand blood oxygen diffusion radius, reduce the aggregation and wall attachment of platelets in blood vessels, thereby improving the hypoxic state of peripheral blood vessels, peripheral nerves, and gonads, promoting angiogenesis and improving microvascularization and circulation, as well as improving the hemodynamics and the oxygen storage capacity of ovarian tissue, enhancing and stabilizing tissue metabolism, and finally, improving ovarian function.

## Conclusion

In summary, for patients undergoing laparoscopic ovarian cystectomy, adjuvant hyperbaric oxygen therapy could significantly improve postoperative ovarian reserve function in patients with remarkable effects. Meanwhile, in the present study, the changes between the control and observation groups were primarily discussed. In the observation group, the intraoperative suturing techniques and matching of Chinese patent medicines were adopted. Whether ovarian function could be improved has not been further investigated. The sample size in the present study was relatively small, and all patients were not followed up for a long period. Therefore, the results of the present study should still be confirmed by a large sample, high-quality, multicenter randomized controlled study.

## Data Availability

The raw data supporting the conclusions of this article will be made available by the authors, without undue reservation.

## References

[1] Van Voorhis BJ Greensmith JE Dokras A Sparks AE Simmons ST Syrop CH. Hyperbaric oxygen and ovarian follicular stimulation for in vitro fertilization: a pilot study. Fertil Steril. (2005) 83(1):226–8. 10.1016/j.fertnstert.2004.05.101 15652917

[2] Duan W Cheng Y. Sequential therapy for kidney-tonifying via traditional Chinese medicine effectively improves the reproductive potential and quality of life of women with decreased ovarian reserve: a randomized controlled study. Am J Transl Res. (2021) 13(4):3165–73. PMID: ; PMCID: 34017485 PMC8129248

[3] Xu XH Chen YC Xu YL Garcinone E blocks autophagy through lysosomal functional destruction in ovarian cancer cells. World J Tradit Chin Med. (2021) 7(2):209–16. 10.4103/wjtcm.wjtcm_81_20

[4] Matsuhashi T Matsui R Hasegawa C Hatori T Kamoi S Takeshita T. Laparoscopic excision of a uterine adenomatoid tumor and a coexisting ovarian teratoma: a case report and literature review. J Nippon Med Sch. (2017) 84(3):139–43. 10.1272/jnms.84.139 28724848

[5] Yuan H Wang C Wang D Wang Y. Comparing the effect of laparoscopic supracervical and total hysterectomy for uterine fibroids on ovarian reserve by assessing serum anti-mullerian hormone levels: a prospective cohort study. J Minim Invasive Gynecol. (2015) 22(4):637–41. 10.1016/j.jmig.2015.01.025 25653041

[6] Li CZ Liu B Wen ZQ Sun Q. The impact of electrocoagulation on ovarian reserve after laparoscopic excision of ovarian cysts: a prospective clinical study of 191 patients. Fertil Steril. (2009) 92(4):1428–35. 10.1016/j.fertnstert.2008.08.071 18930212

[7] Li Q Zhang XQ Ao HF Effects of oxygen pressure exposure on hormone levels and expression of TGF-β1 and Smad3 in chronic stress-induced ovarian dysfunction model rats. Mil Med J South China. (2019) 33(6):371–5. 10.13730/j.issn.1009-2595.2019.06.001

[8] Wang CL Hao J, Qiu LL Effects of hyperbaric oxygen exposure on ovaries, uterus and serum estradiol in rats with premature ovarian failure. Chin J Nautical Med Hyperb Med. (2014), 21(3):145–9. 10.3760/cma.j.issn.1009-6906.2014.03.001

[9] Li S Lin H Xie Y Jiao X Qiu Q Zhang Q. Live births after in vitro fertilization with fertility-sparing surgery for borderline ovarian tumors: a case series and literature review. Gynecol Obstet Invest. (2019) 84(5): 445–54. 10.1159/000497203 30799426

[10] Cerovac A Habek D Hudić I Kamerić L. Laparoendoscopic ovarian-sparing surgery of adnexal tumors in children and adolescents by general gynecologists: a 10-year, retrospective cohort study. J Laparoendosc Adv Surg Tech A. (2021) 31(9):1055–60. 10.1089/lap.2020.0894 34252315

[11] Qiong-Zhen R Ge Y Deng Y Qian ZH Zhu WP. Effect of vasopressin injection technique in laparoscopic excision of bilateral ovarian endometriomas on ovarian reserve: prospective randomized study. J Minim Invasive Gynecol. (2014) 21(2):266–71. 10.1016/j.jmig.2013.07.024 24075865

[12] Asgari Z Rouholamin S Hosseini R Sepidarkish M Hafizi L Javaheri A. Comparing ovarian reserve after laparoscopic excision of endometriotic cysts and hemostasis achieved either by bipolar coagulation or suturing: a randomized clinical trial. Arch Gynecol Obstet. (2016) 293(5):1015–22. 10.1007/s00404-015-3918-4 26493551

[13] Angioli R Muzii L Montera R Damiani P Bellati F Plotti F Feasibility of the use of novel matrix hemostatic sealant (FloSeal) to achieve hemostasis during laparoscopic excision of endometrioma. J Minim Invasive Gynecol. (2009) 16(2):153–6. 10.1016/j.jmig.2008.11.007 19138574

[14] Chun S Ji YI. Effect of hysterectomy on ovarian reserve in the early postoperative period based on the type of surgery. J Menopausal Med. (2020) 26(3):159–64. 10.6118/jmm.20010 33423404 PMC7797220

[15] Fan H He J Bai Y Baicalin improves the functions of granulosa cells and the ovary in aged mice through the mTOR signaling pathway. J Ovarian Res. (2022) 15(1):34. 10.1186/s13048-022-00965-7 35300716 PMC8932175

[16] Zhou Q Tao J Song H Chinese herbal medicine Kuntai capsule for treatment of menopausal syndrome: a systematic review of randomized clinical trials. Complement Ther Med. (2016) 29:63–71. 10.1016/j.ctim.2016.09.011 27912959

[17] Liu W Zhang C Wang L Successful reversal of ovarian hyperstimulation syndrome in a mouse model by rapamycin, an mTOR pathway inhibitor. Mol Hum Reprod. (2019) 25(8):445–57. 10.1093/molehr/gaz033 31329230

[18] Zhang J Fang L Shi L Protective effects and mechanisms investigation of Kuntai capsule on the ovarian function of a novel model with accelerated aging ovaries. J Ethnopharmacol. (2017) 195:173–81. 10.1016/j.jep.2016.11.014 27845267

[19] Lou HP Han WL Ai LL Effect of hyperbaric oxygen combined with drugs on perimenopausal syndrome. Chin J Nautical Med Hyperb Med. (2016) 23(2):164–5. 10.3760/cma.j.issn.1009-6906.2016.02.027

[20] Liu JY. Clinical observation of 29 cases of premature ovarian failure treated with hyperbaric oxygen. Chin J Nautical Med Hyperb Med. (2020) 27(4): 510–2. 10.3760/cma.j.cn311847-20200326-00115

[21] Pineda JF Ortiz CG Moguel Gde J Lopez CR Alcocer HM Velasco ST. Improvement in serum anti-müllerian hormone levels in infertile patients after hyperbaric oxygen (preliminary results). JBRA Assist Reprod. (2015) 19(2):87–70. 10.5935/1518-0557.20150019 27206094

[22] Svensson JF Larsson A Uusijärvi J von Sivers K Kaiser S. Oophoropexy, hyperbaric oxygen therapy, and contrast-enhanced ultrasound after asynchronous bilateral ovarian torsion. J Pediatr Surg. (2008) 43(7):1380–4. 10.1016/j.jpedsurg.2008.02.024 18639702

